# CD4 CAR-T cells targeting CD19 play a key role in exacerbating cytokine release syndrome, while maintaining long-term responses

**DOI:** 10.1136/jitc-2022-005878

**Published:** 2023-01-02

**Authors:** Camilla Bove, Silvia Arcangeli, Laura Falcone, Barbara Camisa, Rita El Khoury, Beatrice Greco, Anna De Lucia, Alice Bergamini, Attilio Bondanza, Fabio Ciceri, Chiara Bonini, Monica Casucci

**Affiliations:** 1 Innovative Immunotherapies Unit, IRCCS Ospedale San Raffaele, Milan, Italy; 2 Experimental Hematology Unit, IRCCS Ospedale San Raffaele, Milan, Italy; 3 Department of Gynecologic Oncology, IRCCS Ospedale San Raffaele, Milan, Italy; 4 Department of Hematology and Stem Cell Transplantation, IRCCS Ospedale San Raffaele, Milan, Italy; 5 Vita-Salute San Raffaele University, Milan, Italy

**Keywords:** Receptors, Chimeric Antigen, CD4-Positive T-Lymphocytes, CD8-Positive T-Lymphocytes, Immunotherapy, Cytokines

## Abstract

**Background:**

To date, T cells redirected with CD19-specific chimeric antigen receptors (CAR) have gained impressive success in B-cell malignancies. However, treatment failures are common and the occurrence of severe toxicities, such as cytokine release syndrome (CRS), still limits the full exploitation of this approach. Therefore, the development of cell products with improved therapeutic indexes is highly demanded.

**Methods:**

In this project, we investigated how CD4 and CD8 populations cooperate during CD19 CAR-T cell responses and what is their specific role in CRS development. To this aim, we took advantage of immunodeficient mice reconstituted with a human immune system (HuSGM3) and engrafted with the B-cell acute lymphoblastic leukemia cell line NALM-6, a model that allows to thoroughly study efficacy and toxicity profiles of CD19 CAR-T cell products.

**Results:**

CD4 CAR-T cells showed superior proliferation and activation potential, which translated into stronger stimulation of myeloid cells, the main triggers of adverse events. Accordingly, toxicity assessment in HuSGM3 mice identified CD4 CAR-T cells as key contributors to CRS development, revealing a safer profile when they harbor CARs embedded with 4-1BB, rather than CD28. By comparing differentially co-stimulated CD4:CD8 1:1 CAR-T cell formulations, we observed that CD4 cells shape the overall expansion kinetics of the infused product and are crucial for maintaining long-term responses. Interestingly, the combination of CD4.BBz with CD8.28z CAR-T cells resulted in the lowest toxicity, without impacting antitumor efficacy.

**Conclusions:**

Taken together, these data point out that the rational design of improved adoptive T-cell therapies should consider the biological features of CD4 CAR-T cells, which emerged as crucial for maintaining long-term responses but also endowed by a higher toxic potential.

**WHAT IS ALREADY KNOWN ON THIS TOPIC**

- Recent clinical data support the key role of CD4 chimeric antigen receptors (CAR)-T cells targeting CD19 in maintaining long-term antitumor responses. However, the relative contribution of CD4 and CD8 CAR-T cell subsets to cytokine release syndrome (CRS) development has not been thoroughly investigated yet.

**WHAT THIS STUDY ADDS**

- By employing SGM3 mice reconstituted with a human immune system and engrafted with the B-cell acute lymphoblastic leukemia cell line NALM-6, we observed that CD4 CAR-T cells are highly prone to activate myeloid cells to induce severe CRS, especially when including the CD28 costimulatory domain. Interestingly, formulating CD4.BBz with CD8.28z CD19 CAR-T cells displayed the highest therapeutic index thanks to a safer toxic profile.

**HOW THIS STUDY MIGHT AFFECT RESEARCH, PRACTICE OR POLICY**

- Our work suggests that the development of new products designed on the CD4 compartment could improve the therapeutic index of current CD19 CAR-T cell therapies.

## Background

So far chimeric antigen receptors (CAR)-T cell therapy has achieved impressive clinical success for the treatment of B-cell acute lymphoblastic leukemia (ALL), non-Hodgkin’s B-cell lymphomas (NHL) and multiple myeloma (MM). These results have led to the approval by the US Food and Drug Administration and the European Medicines Agency of six CAR-T cell products targeting either CD19 or b-cell maturation antigen (BCMA).^1–6^ However, treatment failures due to disease relapse or primary resistance in certain tumor types still represent major concerns.^7–9^ In addition, the occurrence of severe toxicities, such as cytokine release syndrome (CRS) and immune effector cell-associated neurotoxicity syndrome (ICANS), still limits the full exploitation of CAR-T cell therapies.^10–12^ Overall, this picture highlights the need to develop CAR-T cell products with an improved therapeutic index.

Poor CAR-T cell fitness, which refers to the ability of CAR-T cells to expand, persist and exert effector functions after infusion, represents one of the main reasons for treatment failure in patients.^13–15^ These features are influenced by multiple factors, including the composition of the infused product, both in terms of T-cell memory differentiation^16–18^ and CD4/CD8 ratio.^19–22^ Moreover, the use of heterogeneous bulked CAR-T cell formulations greatly limits the possibility of making correlations across different studies, hindering the identification of biomarkers predictive of T-cell expansion, persistence and consequent/potential adverse events. Therefore, procedures able to mitigate product heterogeneity are highly demanded. In this regard, promising results have been obtained either at the preclinical^23^ and clinical level^24 25^ with the administration of CAR-T cells formulated at a defined 1:1 CD4:CD8 ratio, showing improvements especially in terms of safety. In fact, in the context of relapsed/refractory NHL, CD4:CD8 CAR-T cells showed comparable antitumor activity but reduced toxicity, especially severe CRS, compared with unselected commercial products.^3^ These positive results brought to the recent commercialization of the first CD4:CD8 CD19 CAR-T cell product (lisocabtagene maraleucel).^3 26^

From a biological point of view, the contribution of individual CD4 and CD8 CAR-T cell populations in antitumor efficacy remains controversial, while their relative role in toxicity development has not been elucidated yet. To address these unsolved issues, we took advantage of hematopoietic stem/precursor cell (HSPC)-humanized immunodeficient mice (HuSGM3) that, compared with standard xenograft models, recreate the complexity of the interactions among human immune cells, including the myeloid compartment.^27 28^ This crosstalk on one hand supports CAR-T cell expansion and antitumor responses, on the other allows to exacerbate toxicities such as CRS and ICANS, closely recapitulating the clinical behavior of CAR-T cell products. With this model, we have recently demonstrated the superior efficacy and safety profile of CAR-T cells generated from naïve/stem memory subsets rather than total T lymphocytes, supporting the robustness of this model to profile human CAR-T cell performances in mice.^28^ In this study, toxicity assessment in HuSGM3 mice engrafted with the B-ALL cell line NALM-6 revealed that CD4 CAR-T cells targeting CD19 play a crucial role during CRS development, which was more severe when they included the CD28 endodomain rather than 4-1BB. In addition, CD4 CAR-T cells proved able to mediate potent antitumor responses and shape the overall expansion kinetics of the infused product. Finally, we identified a CD4:CD8 formulation endowed with a safer toxic profile, suggesting that the rational combination of these cell subsets may lead to the design of CD19 CAR-T cell products with an improved therapeutic index.

## Methods

### Cell lines

B-ALL leukemic cell lines NALM-6 and BV173 were purchased from the American Type Culture Collection and cultured in Roswell Park Memorial Institute (RPMI) 1640 (Euroclone) supplemented with 10% fetal bovine serum (FBS) (Euroclone), 100 IU/mL penicillin/streptomycin (Euroclone) and glutamine (Euroclone). ALL-CM cell line was kindly provided by Professor Fred Falkenburg (Leiden University Medical Center) and kept in culture in X-VIVO (Euroclone) with 3% human serum (Euroclone) and 100 IU/mL penicillin/streptomycin. For in vivo experiments, NALM-6 cells were transduced with a bidirectional lentiviral vector (LV) including the Gaussia luciferase LUCIA (InvivoGen) in sense and the low-affinity nerve growth factor receptor (LNGFR) selection marker in antisense (Lucia+/NGFR+/NALM-6), as previously reported.^29^ About 293 T cells were used as packaging line for LV production and cultured in IMDM medium (Iscove’s Modified Dulbecco’s Medium) supplemented with 10% FBS, 1% penicillin/streptomycin (100 U/mL, 0,1 mg/mL, Euroclone) and 1% glutamine (2 mM, Euroclone).

### Transduction and culture conditions

Peripheral blood mononuclear cells (PBMCs) were isolated by Ficoll-Hypaque (Lymphoprep, Fresenius) gradient separation. T cells were isolated using the Pan T-cell isolation kit (Miltenyi), CD4 and CD8 fractions were selected with CD4 and CD8 Microbeads (Miltenyi) and stimulated with MACS-GMP T-Cell TransAct (Miltenyi). Both subsets were transduced with a bidirectional LV encoding for either a CD19.CAR.28z or a CD19.CAR.BBz in sense and the truncated LNGFR (ΔLNGFR marker gene in antisense and kept in culture in TeXmacs medium (Miltenyi) supplemented with interleukin (IL)-7 and IL-15 (Miltenyi). Untransduced (UT) CD4 and CD8 T cells were employed as control and separately kept in culture. CAR+T cells were enriched by sorting through magnetic labeling of ΔLNGFR. Phenotypic and functional analyses were performed at the end of manufacturing.

### In vitro functional assays

CD4 and CD8 CAR-T cells alone or combined in a defined CD4:CD8 composition were tested in functional assays. For lytic activity, CAR-T cells were co-cultured with CD19+ leukemic cell lines (Lucia+/NGFR+/NALM-6; ALL-CM; BV173) at different effector:target (E:T) ratios. UT CD4, CD8 or CD4:CD8 T cells were employed as controls. After 24 hours, supernatants were collected and analyzed with the LEGENDplex bead-based cytokine immunoassay (BioLegend). After 4 days, residual cells in culture were analyzed by fluorescence activated cell sorting (FACS) using Flow-Count Fluorospheres (Beckman Coulter). Tumor cell killing, expressed as elimination index, was calculated as follows: 1—(number of residual target cells in presence of CD19 CAR-T cells/number of residual target cells in presence of UT T cells). For proliferation assay, CAR-T cells were co-cultured with CD19+ tumor cells at the 1:1 E:T ratio. After 4 days, cells were analyzed for intracellular Ki-67 by FACS. For activation assay, CAR-T cells were co-cultured with CD19+ tumor cells at the 1:3 E:T ratio. After 24 hours, cells in culture were analyzed by FACS and stained for CD69 and CD25 expression. Finally, tripartite co-cultures comprizing CAR-T cells, NALM-6 leukemia and autologous monocytes or THP-1 monocyte-like cells were performed at a 1:1 E:T ratio. After 24 hours, supernatants were collected and analyzed for cytokine detection. CAR-T cell apoptosis was analyzed 48 hours after thawing by Annexin V (BioLegend) and 7-AAD (BioLegend) staining. Antigen-independent proliferation was investigated taking advantage of CellTrace Proliferation Kit (Thermo Fisher Scientific) and analyzed by FACS 7 days after culture.

### In vivo functional assays

Mice, 6–8 weeks old, NSGTgCMV-IL3, CSF2, KITLG1Eav/MloySzJ (SGM3) were sublethally irradiated and infused intravenous with 1×10^5^ human cord blood (CB) CD34+ cells. These cells were either purchased (Lonza) or purified from umbilical CB samples, thanks to the collaboration with the Gynecology Unit at OSR (Protocol 34CB). On reconstitution, HuSGM3 mice were infused with 0.5×10^6^ Lucia+/NGFR+/NALM-6 cells and 5 or 7 days after treated with CAR-T cells or UT T cells, according to efficacy and toxicity setting, respectively. Mice were sacrificed when relative bioluminescent units exceeded the threshold of 1×10^6^ or when manifesting clinical signs of suffering. For evaluating CRS development, weight loss was daily monitored, and the concentration of serum human cytokines (LEGENDplex, BioLegend) was assessed at day 4, according to manufacturer instructions. CRS incidence and grading were calculated by considering multiple parameters (ie, weight loss, mice death, together with IL-6, monocyte chemoattractant protein-1 (MCP-1) and interferon gamma-induced protein-10 (IP-10) myelo-derived cytokines). The overall CRS score resulted from the sum of the scores associated to each parameter, which were pondered according to the level of statistical significance occurring between deaths related to severe CRS and recovering animals, as previously described.^28^

### Flow cytometry

For all the experiments CAR-T cells, tumor lines and mouse samples were stained with one or more monoclonal antibodies listed in online supplemental material. Flow-cytometry data were acquired using BD Canto II cell analyzers and visualized with FlowJo V.10.8.1 software.

10.1136/jitc-2022-005878.supp1 Supplementary data

### Statistics

Statistical analyses were performed with Prism Software V.9.4.1 (GraphPad). Data are shown as mean±SEM with at least n=3 replicates and at least from two independent healthy donors. Data sets were analyzed with paired or unpaired Student’s t-test, two-way analysis of variance, or Ghean-Breslow-Wilcoxon and Mantel-Cox two-sided log-rank tests depending on the experimental design. Differences with a p value<0.05 were considered as statistically significant.

## Results

### CD4 CAR-T cells are less lytic but show superior proliferation and activation levels

**Figure 1:**
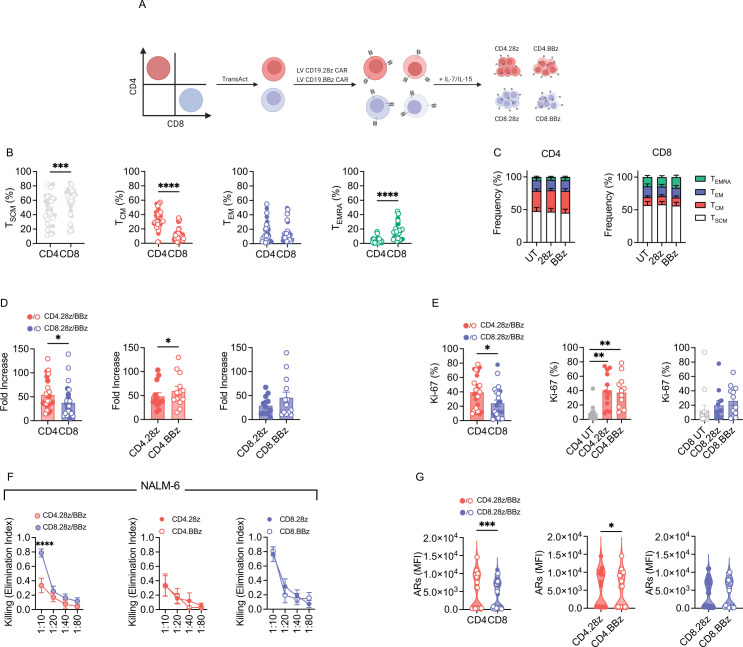
CD4 CAR-T cells display greater activation and proliferation potential. (A) Schematic of CD4 and CD8 CAR-T cell manufacturing. CD4 and CD8 subsets were selected through magnetic sorting, activated, transduced with a lentiviral vector encoding CD19.28z or CD19.BBz CARs and expanded with IL-7/IL-15. (B) Memory phenotype at the end of manufacturing (n=16). (C) Memory phenotype at the end of manufacturing based on CAR co-stimulation (n=8). Untransduced (UT) CD4 and CD8 T cells were used as controls. (D) CAR-T cell fold expansion at the end of manufacturing (n=13). (E) CAR-T cell proliferation after 4-day co-culture with CD19+ tumor cells measured by intracellular staining of Ki-67 (n=3 donors against BV173 and ALL-CM, n=6 donors against NALM-6). (F) Killing activity expressed as Elimination Index and measured after 4-day co-culture with tumor cells at different effector/target (E:T) ratios (n=8). (G) CAR-T cell activation measured as mean fluorescence intensity (MFI) of CD69 and CD25 activation receptors (ARs) 1 day after co-culture with tumor cells (n=3). Data are represented as mean±SEM with overlapping scattered values. *p<0.05, **p<0.01, ***p<0.001, ****p<0.0001 by paired t-test or two-way analysis of variance. CAR, chimeric antigen receptors; IL, interleukin; T_CM_, central memory T cells; T_SCM_, stem memory; T_EM_, effector memory; T_EMRA_, effector subtypes.

To profile CD4 and CD8 CAR-T cells activity embedded with different co-stimulatory domains, we collected CD3+ T cells from PBMCs and magnetically sorted CD4+ and CD8+ T cells. Both CD4 and CD8 fractions were activated with a polymeric nanomatrix, transduced with LV encoding for a CD19 CAR harboring either CD28 or 4-1BB endodomains and expanded with IL-7 and IL-15, according to a protocol that better preserves T-cell fitness (figure 1A). Transduced cells were selected based on the expression of the ΔLNGFR marker gene and UT CD4 and CD8 populations were employed as controls.

Phenotypic characterization of the cell products revealed superimposable ΔLNGFR expression levels (online supplemental figure 1A, B and B) and some degrees of heterogeneity in the memory differentiation status (figure 1B). While CD4 cell products contained higher proportions of central memory T cells, CD8 cell products were enriched in stem memory and effector subtypes (figure 1B). Interestingly, memory differentiation within each T-cell lineage was unaffected by the CAR co-stimulus and was comparable to the UT counterpart (figure 1C). Superior expansion during manufacturing was characteristic of CD4 CAR-T cells, especially with the 4-1BB design (figure 1D). This behavior was maintained on antigen encounter in co-culture experiments with CD19+ tumor cells (figure 1E). Conversely, as expected, lytic activity was better supported by CD8 CAR-T cells against aggressive NALM-6 cells (figure 1F), while not significant differences were appreciated against slow-growing targets (online supplemental figure 1C, D). Interestingly, CD4 CAR-T cells, especially those including CD28, displayed increased activation levels after antigen encounter compared with CD8 CAR-T cells (figure 1G). However, further characterization of the final product revealed no signs of tonic signaling after thawing, as indicated by negligible expression of exhaustion markers (online supplemental figure 1E), low apoptosis (online supplemental figure 1F) and lack of antigen-independent proliferation (online supplemental figure 1G).

Collectively, these data show that CD4 CAR-T cells are characterized by greater activation levels after antigen encounter and increased proliferative capacity, while CD8 CAR-T cells feature improved cytotoxicity.

### CD4 CAR-T cells induce higher monocyte activation and cytokine release

**Figure 2:**
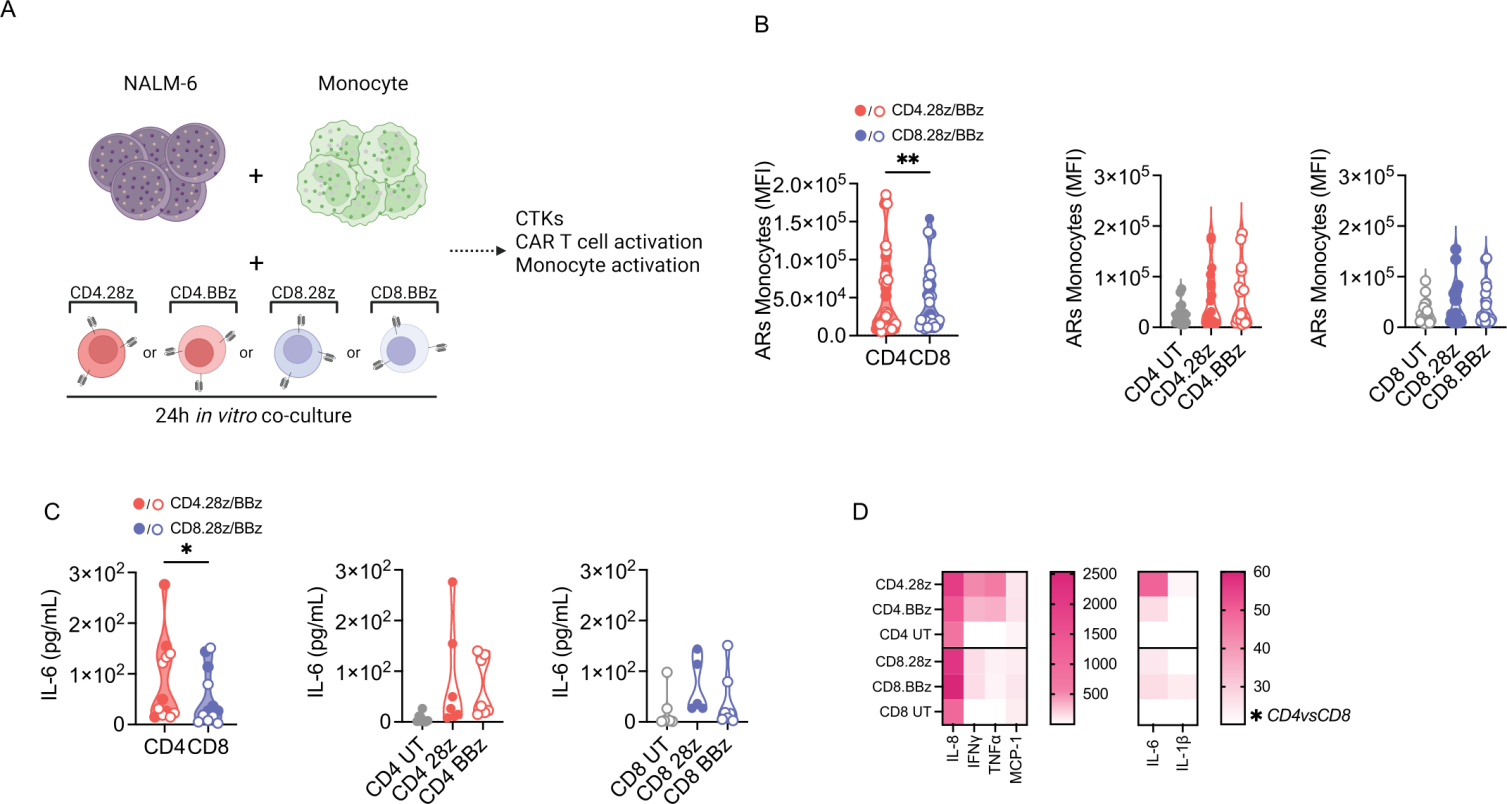
CD4 CAR-T cells are more potent in triggering monocyte activation and cytokine release. (A) Schematic of tripartite co-cultures including NALM-6 leukemia cells, CAR-T cells and autologous monocytes. UT CD4 and CD8 T cells were used as controls. CTKs, cytokines. (B) Activation receptors upregulation (ARs: CD54, CD86, HLA-DR) on monocytes expressed as MFI 1 day after plating (n=6). (C) IL-6 production and (D) heatmap visualization of cytokine release 1 day after plating. Data are represented as mean±SEM or mean±SEM with overlapping scattered or scaled values according to a graded-color range depending on relative minimum and maximum levels, when referring to the heatmap. *p<0.05, **p<0.01, by paired t test or two-way analysis of variance. HLA-DR, human leukocyte antigen-DR isotype; IFN, interferon; IL, interleukin; MFI, mean fluorescence intensity; TNF, tumor necrosis factor; UT, untransduced.

After in vitro functional validation of our CAR-T cell products, we got interested in deciphering the contribution of individual CD4 and CD8 CAR-T cells in triggering detrimental toxicities, particularly CRS, in B-ALL context. To address this issue, we employed tripartite in vitro co-cultures comprizing NALM-6 leukemia, CD19 CAR-T cells and autologous monocytes (figure 2A), as previously described.^28^ Of note, we observed increased monocyte activation levels when including CD4 rather than CD8 CAR-T cells, independently of the endodomain incorporated (figure 2B). Accordingly, also IL-6 production (figure 2C) and release of other inflammatory cytokines (figure 2D) were greater in the presence of CD4 CAR-T cells. Similar results were achieved when employing the THP-1 cell line instead of primary monocytes (online supplemental figure 2).

Collectively, these findings suggest that CD4 CAR-T cells play a pivotal role in tuning monocyte responses, both in terms of activation and cytokine release.

### CD4 but not CD8 CAR-T cells exacerbate severe cytokine release syndrome

**Figure 3:**
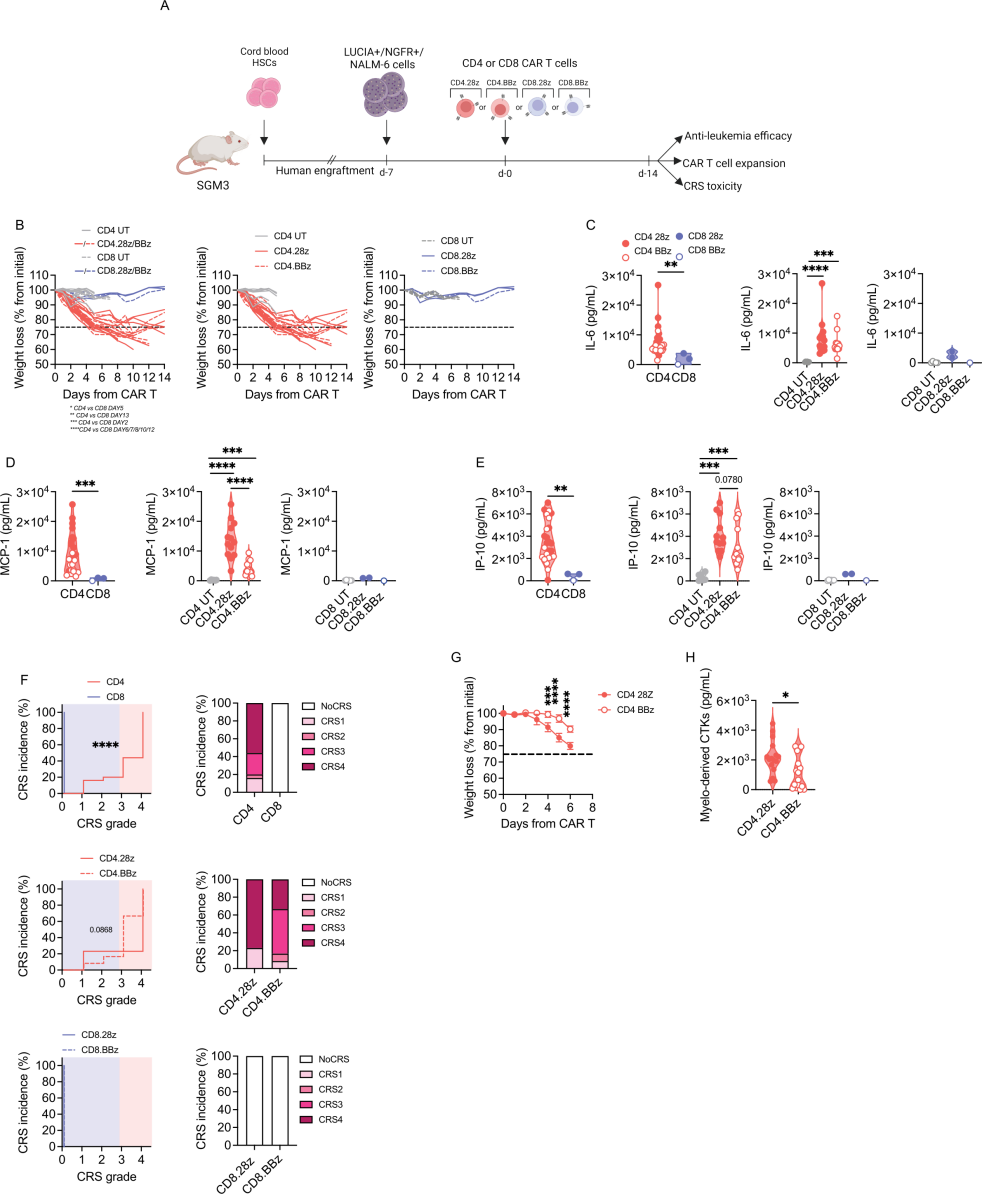
CD4 CAR-T cells exacerbate CRS, especially with the CD28 design. (A) SGM3 mice were reconstituted with human hematopoietic stem/precursor cells (HuSGM3) and injected with Lucia+/NGFR+/NALM-6 leukemia cells. After reaching a high tumor burden, mice were treated with high T-cell doses. Only mice that responded to therapy were included in CRS analysis: CD4.28z (n=13), CD4.BBz (n=12), CD8.28z (n=2) and CD8.BBz (n=1). CD4 UT and CD8 UT (n=6) were used as control. (B) Weight loss at different time points. (C) IL-6, MCP-1 (D) and IP-10 (E) serum levels 4 days after treatment. (F) CRS grading. Left panels: Kaplan-Meier curves. Right panels: Histograms summarizing CRS grading. (G) After reaching a high tumor burden, mice were treated with low doses of CD4.28z and CD4.BBz (n=4) and monitored for weight loss at different time points. (H) Myelo-derived cytokines (IL-6, IP-10, MCP-1) serum levels 4 days after treatment. Data are represented as box and violin plots, mean±SEM together with scaled values according to a graded-color range depending on relative minimum and maximum levels, when referring to the heatmap. *p<0.05, **p<0.01, ***p<0.001, ****p<0.0001, by two-way analysis of variance, unpaired t-test and Gehan-Breslow-Wilcoxon test. CAR, chimeric antigen receptors; CRS, cytokine release syndrome; HSC, hematopoietic stem cell; MCP-1, monocyte chemoattractant protein-; IP-10, interferon γ-induced protein 10; IL, interleukin; UT, untransduced.

To better profile the CRS potential of different cell products, we took advantage of the humanized mouse model developed in our unit.^28^ Briefly, SGM3 mice were reconstituted with human HSPCs, infused with the B-ALL cell line NALM-6 and treated with anti-CD19 CD4 or CD8 CAR-T cells carrying either CD28 or 4-1BB (figure 3A). Interestingly, while leukemia control was achieved in all animals treated with CD4 CAR-T cells, most animals injected with CD8 CAR-T cells succumbed to the disease (online supplemental figure 3A). Such poor antitumor activity possibly reflected expansion failure of both CD8 cell products (online supplemental figure 3B), suggesting that the crosstalk with innate immune components is not sufficient for CD8 to expand and fully exert effector functions, and that CD4 support is required. Since CRS typically occurs in patients who respond to CAR-T cell therapy, we only considered mice that achieved tumor remission for CRS assessment. Surprisingly, the majority of CD4 CAR T cell-treated mice experienced severe and irreversible weight loss, while mice injected with CD8 products did not suffer from any weight reduction (figure 3B). This observation was accompanied by greater elevation of IL-6 (figure 3C), MCP-1 (figure 3D) and IP-10 (figure 3E) in mice that received CD4 CAR-T cells. In accordance, these animals also showed decreased survival related to severe CRS (online supplemental figure 3C). To stratify CRS development more precisely, we used an algorithm that assigns to each mouse a CRS score, recapitulating the grading system employed in the clinic.^28^ This analysis confirmed that high-grade CRS occurred exclusively in mice treated with CD4 CAR-T cells (figure 3F). Interestingly, by clustering CD4 CAR-T cell products based on endo-co-stimulation, we appreciated higher MCP-1 levels in case of CD28 rather than 4-1BB. Accordingly, most of the mice were treated with CD4.28z CAR-T cells experienced grade 4 CRS, while those receiving CD4.BBz CAR-T cells suffered from a less severe CRS. Given the existence of patients who develop CRS after CAR-T cell activation but eventually do not achieve a complete response, we also analyzed CRS parameters in mice that did not respond to CD8 CAR T-cell therapy. Of note, we did not observe any sign of CRS, neither weight loss nor cytokine elevations, in this group of animals (online supplemental figure 3D, E). To further dissect the influence of co-stimulation on CD4 CAR-T cell behavior, we repeated the experiment by lowering the T-cell dose, in the attempt of uncovering/bringing to light subtle differences. In this setting, neither CD4.28z or CD4.BBz CAR-T cells were able to counteract leukemia growth (online supplemental figure 3F). However, even though monitored for a limited time frame, mice treated with CD4.28z CAR-T cells displayed a greater weight loss compared with CD4.BBz products (figure 3G). In accordance, a superior release of myelo-derived cytokines was observed when CD4.28z CAR-T cells were employed (figure 3H).

Taken together, these results hint that CD4 CAR-T cells are crucial players in CRS development and that CD28 co-stimulation is associated with an increased toxic potential when dealing with the CD4 T-cell lineage.

### Differentially co-stimulated CD4:CD8 CAR-T cells display similar activity in vitro

**Figure 4:**
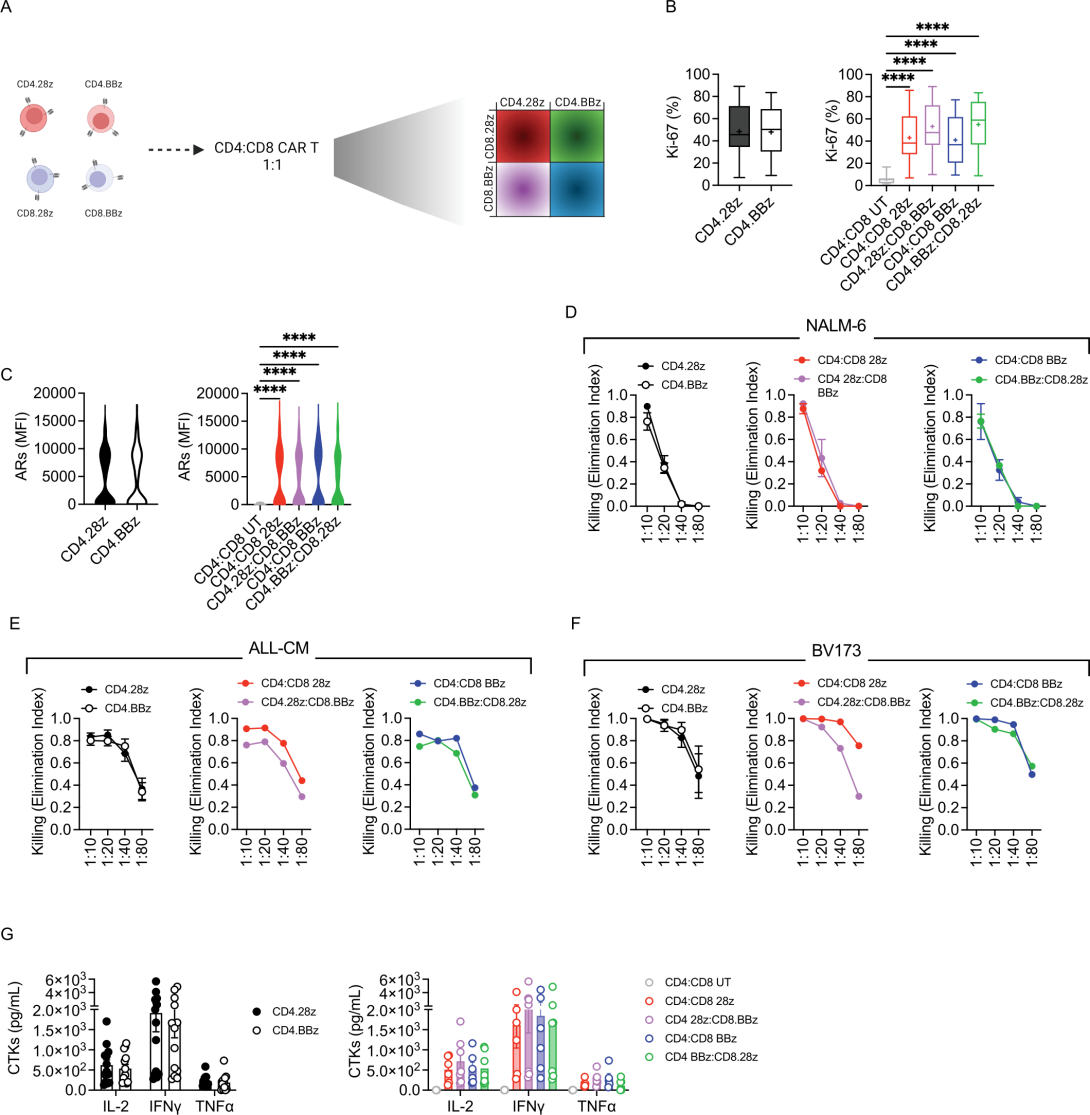
CD4:CD8 CAR-T cells display similar in vitro activity. (A) Schematic of CD4:CD8 CAR-T cell manufacturing. The conditions previously employed were then formulated at a 1:1 ratio as follows: CD4:CD8 28z, CD4:CD8 BBz, CD4.28z:CD8.BBz and CD4.BBz:CD8.28z. (B) CAR-T cell proliferation after 4-day co-culture with tumor cells, measured by intracellular staining of Ki-67 (n=3 donors against BV173, n=4 against ALL-CM and n=6 against NALM-6). UT CD4 and CD8 T cells were used as controls. (C) CAR-T cell activation measured as MFI of CD69 and CD25 ARs 1 day after co-culture with tumor cells (n=3). Killing activity expressed as Elimination Index and measured by co-culturing CAR-T cells with (D) NALM-6 (n=3), (E) ALL-CM (n=4) and (F) BV173 (n=3) tumor cells for 4 days at different E:T ratios. (G) Cytokine production after 24-hour co-culture of T cells with tumor cells at a 1:10 E:T ratio (n=3 donors against NALM-6 and ALL-CM, n=1 against BV173). Data are represented as mean±SEM with overlapping scattered values. ****p<0.0001 by paired t-test or two-way analysis of variance. ARs, activation receptors; CAR, chimeric antigen receptors; CTKs, cytokines; E:T, effector:target; IFN, interferon; IL, interleukin; MFI, mean fluorescence intensity; TNF, tumor necrosis factor; UT, untransduced.

Once completing the assessment of the individual subsets, we extended our studies to experimental groups where differentially co-stimulated CD4 and CD8 CAR-T cells targeting CD19 were formulated in a defined 1:1 ratio (figure 4A). Based on our previous results, we decided to maintain the focus on CD4 CAR-T cells, to evaluate whether their activity could be modified when combined with different CD8 populations. Among the treatment groups, no significant differences could be appreciated in terms of proliferative abilities, despite differentially co-stimulated combinations displaying a slightly superior proliferation (figure 4B). Concordantly, activation levels were similar among all the formulations (figure 4C). In addition, as expected, we observed comparable cytotoxic activity of all groups challenged either against fast-growing (figure 4D) or slow-growing (figure 4E, F) tumor cells. This behavior was also reflected by similar cytokine release on antigen encounter in vitro (figure 4G).

Overall, in vitro testing revealed no major differences between differentially co-stimulated CD4:CD8 CAR-T cell products.

### CD4.BBz:CD8.28z CAR-T cells displays slightly reduced CRS incidence and severity

**Figure 5:**
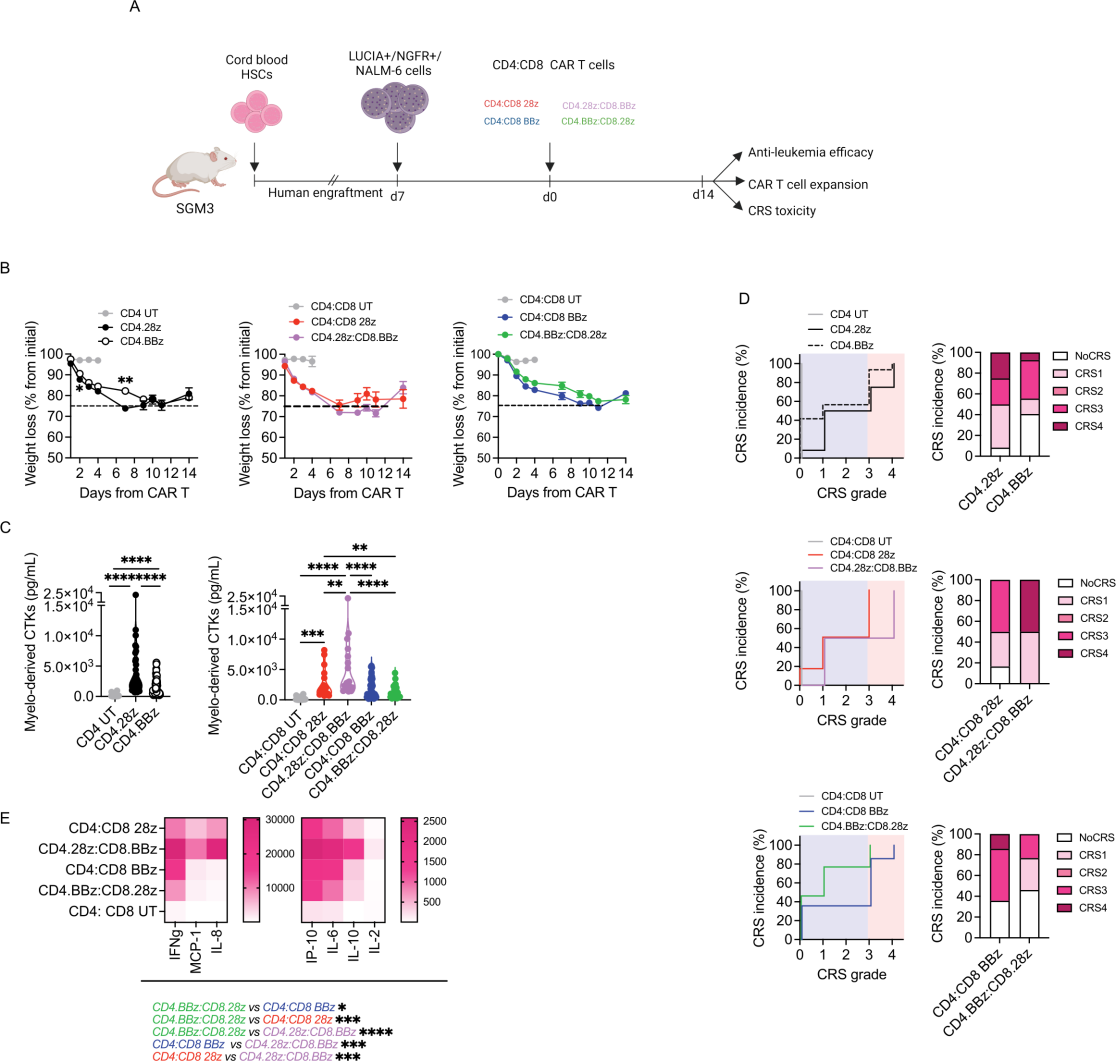
CD4.BBz:CD8.28z displays slightly reduced CRS incidence and severity. (A) SGM3 mice were reconstituted with human hematopoietic stem/precursor cells (HuSGM3) and injected with Lucia+/NGFR+/NALM-6 leukemia cells. After reaching a high tumor burden, mice were treated with high doses of CD4:CD8 UT (n=8), CD4:CD8 28z (n=6), CD4:CD8 BBz (n=6), CD4.28z:CD8.BBz (n=14), CD4.BBz:CD8.28z (n=13). (B) Weight loss at different time points. (C) Myelo-derived cytokine (IL-6, IP-10, MCP-1) serum levels 4 days after treatment. (D) CRS grading. Left panels: Kaplan-Meier curves. Right panels: Histograms summarizing CRS grading. (E) Heatmap visualization of serum cytokine levels 4 days after CAR-T cells infusion. Data are represented as mean±SEM with overlapping scattered values and box and violin plots. *p<0.05, **p<0.01, ***p<0.001, ****p<0.0001, by two-way analysis of variance, unpaired t-test and Gehan-Breslow-Wilcoxon test. CAR, chimeric antigen receptors; CRS, cytokine release syndrome; CTKs, cytokines; HSC, hematopoietic stem cell; MCP-1, monocyte chemoattractant protein-; IP-10, interferon γ-induced protein 10; IFN, interferon; IL, interleukin; UT, untransduced.

Next, we moved into testing the potential of these different cell formulations to exacerbate CRS in the animal model mentioned before (figure 5A). In this setting, all treatments displayed similar antitumor activity (online supplemental figure 4A). Concerning toxic potential, differences between groups were not easy to capture and severe CRS-related survival rates were superimposable among all the treated mice (online supplemental figure 4B). However, in line with previous results, we noticed that formulations including CD4.28z were characterized by more severe weight loss (figure 5B, left) and higher myelo-derived cytokine levels (figure 5C, left), as compared with products comprizing CD4.BBz. This resulted in a higher incidence of grade 4 CRS and only a minimal fraction of mice that remained CRS-free (figure 5D, left). When looking at each product individually, we observed that differently co-stimulated CAR-T cell products are positioned at the two extremes, with CD4.28z:CD8.BBz being the most toxic and CD4.BBz:CD8.28z the least (figure 5C, D, middle and right). Accordingly, CD4.BBz:CD8.28z cells were less prone to generate a cytokine storm over all the other conditions (figure 5E), especially when looking at those formulations including CD4.28z.

Overall, these data remark the contribution of CD4.28z during CRS development and suggest a formulation (CD4.BBz:CD8.28z) associated with a lower toxic potential.

### Overall expansion kinetic and persistence in vivo are guided by CD4 CAR-T cells

**Figure 6:**
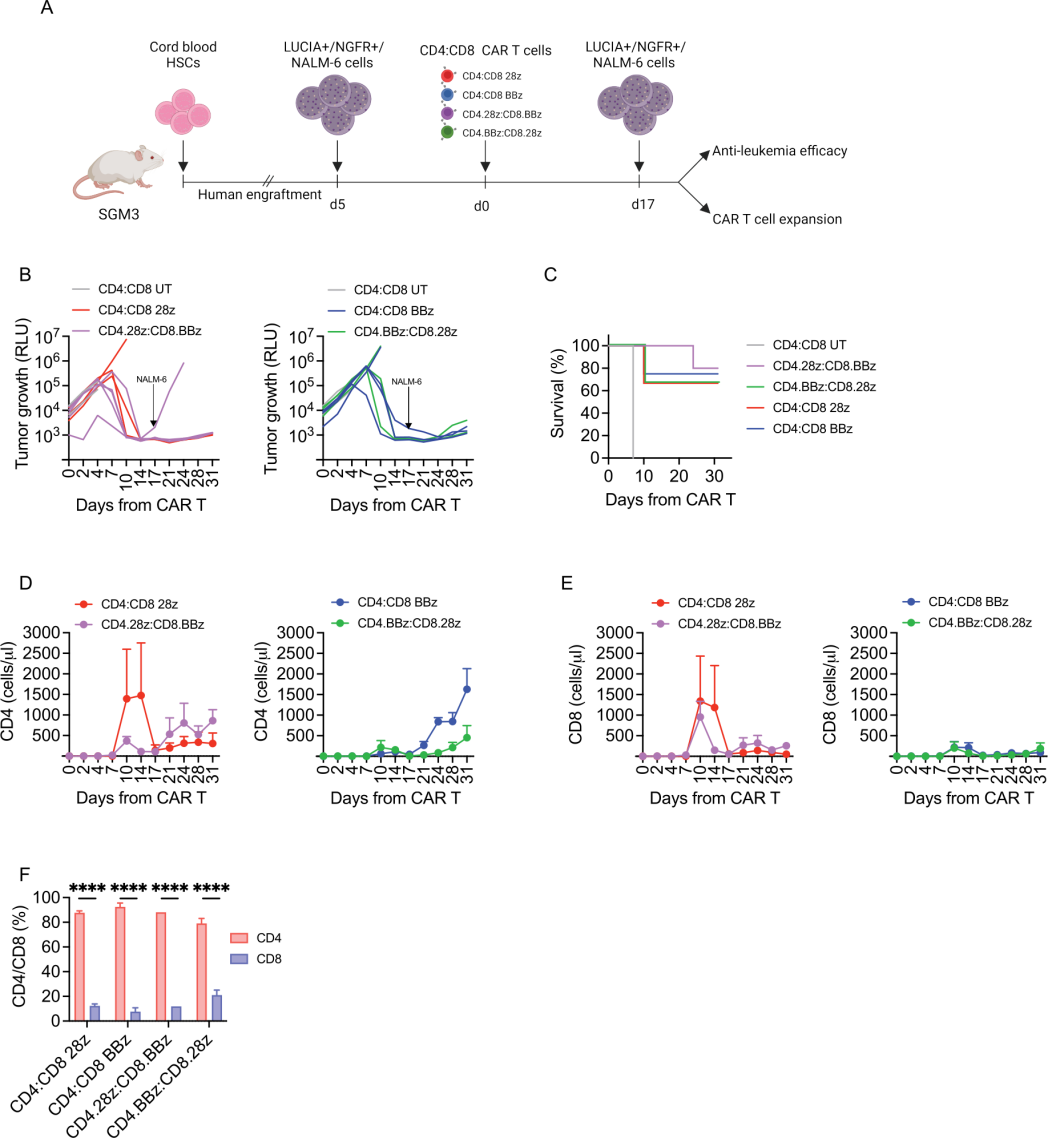
Expansion profile and kinetic are guided by CD4 CAR-T cells. (A) Schematic of HuSGM3 injected with Lucia+/NGFR+/NALM-6 leukemia cells and treated with low doses of CD4:CD8 UT (n=2), CD4:CD8 28z (n=3), CD4:CD8 BBz (n=4), CD4.28z:CD8.BBz (n=5), CD4.BBz:CD8.28z (n=3) after reaching a mid-tumor burden. (B) NALM-6 bioluminescence signal at different time points after treatment. (C) Kaplan-Meier survival analysis. (D) CD4 and (E) CD8 CAR-T cell expansion peak at different time points after treatment. (F) CD4/CD8 frequency in the bone marrow of surviving mice at sacrifice (n=2). Data are represented as mean±SEM with individual and overlapping scattered values. ****p<0.0001, two-way analysis of variance, by unpaired t-test and Mantel-Cox 2-sided log-rank test. CAR, chimeric antigen receptors; HSC, hematopoietic stem cell; RLU, relative bioluminescent units; UT, untransduced.

To gain insights into the therapeutic index of the different CD19 CAR-T cell products, we challenged their antitumor activity in the humanized mouse model. To this aim, we employed low CAR-T cell doses and performed NALM-6 rechallenge at the end of the first response (figure 6A). Even in this challenging setting, all groups were equally able to counteract leukemia growth (figure 6B) and no significant differences were observed in terms of leukemia-related survival rates (figure 6C), recapitulating our previous in vitro results. Strikingly, however, CD4 CAR-T cells were able to shape overall T-cell kinetics, under the influence of the endodomain embedded. Indeed, CD4.28z induced the characteristic peak of expansion associated with CD28 endodomain in both CD4 and CD8 T-cell compartments (figure 6D), while CD4.BBz was associated with delayed proliferation kinetics (figure 6E). Interestingly, this happened independently of the CD8 populations they were mixed with. Finally, in accordance with clinical evidence, we detected a preponderance of CD4 CAR-T cells in the bone marrow of mice who achieved tumor control (figure 6F), supporting their role in the maintenance of long-term antitumor activity.

Overall, these data suggest that differently costimulated CD4:CD8 CAR T-cell products have similar antitumor activity and highlight the crucial role of CD4 CAR-T cells in shaping T-cell proliferation and maintain antitumor responses in vivo.

## Discussion

In this study we have demonstrated that CD4 CAR-T cells targeting CD19 play a crucial role in CRS development, being also actively involved in maintaining antitumor responses within the frame of B-ALL malignancy. Interestingly, we observed that CD4 CAR-T cells including the CD28 endodomain showed a higher CRS potential compared with 4-1BB products, suggesting that tailoring CAR design to a specific cell subset might modulate its behavior, especially in terms of safety. Accordingly, when we tested different 1:1 CD4:CD8 CAR-T cell formulations, despite similar antitumor activity, CD4.BBz:CD8.28z CAR-T cells emerged as the product with the lowest toxic potential.

For a long time, CD4 T cells have been confined to a helper-priming function to support the killing role of CD8 T cells, without claiming intrinsic cytotoxic activity. Physiologically, CD4 T cells play a critical role in licensing dendritic cells to optimize both magnitude and quality of CD8 T-cell responses.^30^ This subset is also implicated in supporting antitumor immunity and is responsible for negative regulation of other effector cells, like CD8 T lymphocytes and macrophages. The different behavior of these two cell subsets is reflected also by intrinsic metabolic differences, as CD4 T cells are known to sustain an oxidative metabolism while CD8 T cells are characterized by a more glycolytic metabolic reprogramming, thus explaining the previous observations.^30 31^

These remarks notwithstanding, in recent years either clinical and preclinical studies put emphasis on the direct involvement of CD4 T cells and especially CAR-T cells in tumor control. For instance, in a cohort of 244 patients with metastatic bladder cancer, intratumoral cytotoxic CD4 T cells with distinct expression of proliferation markers have been identified and correlated with clinical response to anti-programmed death ligand-1 therapy,^32–34^ thus overcoming a mere helper role previously conceived for CD4 T cells. Moreover, a 10-year follow-up of two patients with chronic lymphocytic leukemia who achieved remission after treatment with CD19 CAR-T cells uncovered that the engineered population still present at later time points was dominated by CD4 T lymphocytes. Interestingly, this persisting cell subset exhibited robust activation, potent cytotoxicity and elevated proliferation capacity, thus enforcing a key role for CD4 CAR-T cells in sustaining antileukemia response and long-term remission.^35^ Recently, also preclinical studies have supported the central, pivotal contribution of CD4 CAR-T cells in maintaining antitumor responses. In xenograft mouse models of pleural malignancies and glioblastoma, persisting CAR-T cells demonstrated predominant enrichment in the CD4 compartment, which proved essential to guarantee prolonged efficacy.^36 37^ A transcriptomic analysis suggested a lower susceptibility to exhaustion than CD8 cells might explain their long-lasting antitumor activity, even after multiple rechallenges.^37^ Similarly, in a syngeneic mouse model of CD19 CAR-T cell therapy, it has been reported that CD4 CAR-T cells retain antitumor efficacy despite concomitant T cell receptor stimulation, while CD8 CAR-T cells become exhausted and undergo apoptosis, thus losing antitumor activity.^38^ As highlighted in these studies, also our group gave proof of longer persistence of CD4 rather than CD8 CAR-T cells in both hematologic^28^ and solid tumor contexts.^39^

In the current work, by using different cell products, we confirmed the preferential accumulation of anti-CD19 CD4 CAR-T cells at later time points in the bone marrow of leukemic mice who achieved complete response, strengthening the curative value of this cell subset. Furthermore, interestingly, CD4 CAR-T cells appeared responsible for shaping overall T-cell expansion kinetics in vivo. Indeed, products including CD4.28z exhibited the distinctive CD28-associated T-cell peak, while products harboring CD4.BBz showed elevated expansion rates at later time points, regardless of the endo-co-stimulus provided to CD8 CAR-T cells.

In our in vivo experiments, CD4 CAR-T cells have shown to be extremely efficient in counteracting tumor growth when administered as a single agent, while CD8 CAR-T cells alone have been ineffective in most cases. Conversely, in vitro, CD8 CAR-T cells displayed superior lytic activity than CD4 CAR-T cells, suggesting us to ascribe this controversial behavior to a lower expansion capacity and an insufficiently supportive environment in vivo. The fact that we obtained these results using immunodeficient mice reconstituted with a functional immune system suggests the inadequacy of other immune cells to sustain the proper functioning of CD8 CAR-T cells and the crucial role of CD4 CAR-T cell help. To this regard, literature seems controversial. Many studies concur with our observations, pointing to a superior antitumor potential of CD4 CAR-T cells when administered as single therapy,^36 37 40–42^ while some others sustain the superiority of CD8 CAR-T cells, at least in the context of B-cell malignancies.^23 43^ Such discrepancies can be ascribed to the employment of different mouse models (xenograft vs syngeneic), tumor types (solid vs hematological) and manufacturing procedures (IL-2 vs IL-7/IL-15). Several murine models have been used to model CAR-T cell responses, often leading to divergent results and making the validation of different CAR-T cell products challenging and poorly standardized. Syngeneic murine models are highly relevant as they well represent the complexity of the interactions between the host immune system and CAR-T cells, especially for solid tumor contexts.^44^ However, human CAR-T cell products cannot be evaluated in these models, and discrepancies between human and murine immune systems may represent a limitation for translating results to the clinic.^45^ Immunodeficient mice reconstituted with a human immune system represent a bridge between standard xenograft and syngeneic mouse models. Indeed, they allow testing human CAR-T cell products in mice while ensuring proper crosstalk with other human immune cells and cytokines, which are crucially involved in supporting CAR-T cell function and in exacerbating CAR-T cell-related toxicities, like CRS and ICANS.^27 28 45^ For these reasons, we exploited the HuSGM3 mouse model to evaluate CD4 and CD8 CAR-T cell performances. Among the most interesting data emerging from these analyses, we found the striking potential of CD4 CAR-T cells to cause severe CRS. We previously described that a parameter deeply influencing the severity of toxic manifestations is CAR-T cell activation potential, which modulates triggering of myeloid cells to release inflammatory mediators.^28^ In line with this, our data suggest that CD4 CAR-T cells hold a lower activation threshold compared with CD8 CAR-T cells, which render them more efficient in activating the myeloid compartment. Accordingly, in a syngeneic mouse model of Burkitt-like lymphoma it has been shown that CD4 CAR-T cells are more efficient than CD8 CAR-T cells in recruiting and activating host immune cells, such as NK cells, dendritic cells and monocytes.^43^ As stated above, our data suggest that the toxic potential of CD4 cells is also increased when CD28 co-stimulatory domain, rather than 4-1BB, is included in the CAR design. This observation is in line with recent reports highlighting that CAR-T cells including the CD28 costimulatory domain appear more likely to induce CRS and neurotoxicity in patients than products containing 4-1BB.^46–50^ However, besides costimulatory domains, CD19 CAR-T cell products also differ for several other parameters that can impact toxicity development, such as manufacturing procedure and target populations, challenging the possibility of making direct comparisons on available data. Mechanistically, increased toxic potential could be related to the fact that CD28 endodomain provides accelerated expansion and a stronger intracellular signal than 4-1BB, which in turn renders CD28-costimulated CAR-T cells less sensitive to antigen density.^35 51–53^ Therefore, our data imply that the use of 4-1BB, and presumably similarly acting co-stimulatory domains, in CD4 CAR-T cells could be beneficial to diminish CRS severity. To date, employment of alternative combinations of co-stimulatory domains in CD4 and CD8 cell subsets has been evaluated in terms of efficacy in solid tumors.^54^ However, the CRS potential of these cell products remains to be investigated. Alternatively, transducing CD4 T cells with low-affinity CAR constructs or with CARs targeting antigens expressed at low levels could reduce their activation levels on tumor encounter, thus mitigating the consequent development of lethal toxicities. Notwithstanding these observations, we acknowledge that the translational potential of strategies involving transduction of CD4 and CD8 T cells with different constructs can be laborious and seriously hampered by high production costs. Therefore, an attentive cost-benefit analysis is required before clinical translation.

In conclusion, we provide new insights into the crucial involvement of CD4 CAR-T cells targeting CD19 during antileukemia responses, especially in maintaining long-term efficacy and triggering detrimental toxicities, such as CRS. Our data suggest that the rational design of new products taking into consideration the intrinsic features of CD4 and CD8 T-cell subsets could improve the therapeutic index of current CD19 CAR-T cell therapies of B-cell tumors.

## Data Availability

All data relevant to the study are included in the article or uploaded as supplementary information.
