# Supplementary material for: CD4 CAR-T cells targeting CD19 play a key role in exacerbating cytokine release syndrome, while maintaining long-term responses
Source: J Immunother Cancer. 2023 Jan 2;11(1):e005878. doi: 10.1136/jitc-2022-005878 (PMC9809278; doi:10.1136/jitc-2022-005878)
Supplement: Supplementary data [file jitc-2022-005878supp001.pdf]

BMJ Publishing Group Limited (BMJ) disclaims all liability and responsibility arising from any reliance placed on this supplemental material which has been supplied by the author(s)

Supplementary Material

Supplementary Methods

Antibodies employed for FACS analysis

CD3 PB (Biolegend, cloneHIT3a), CD45 BV510 (Biolegend, clone HI30), CD271 PE-Cy7 (BD, clone CD40-1457), CD271 PE (BD, clone C40-1457), CD4 FITC (Biolegend, clone SK3), anti-mouse CD45 PerCP (Biolegend, clone 30-f11), CD14 APC (Biolegend, clone M5E2), CD19 APC/Cy7 (Biolegend, clone HIB19), CD56 PE-Cy7 (Biolegend, clone HCD56), HLA-DR APC/Cy7 (Biolegend, clone L243), CD45RA FITC (Biolegend, clone HI100), CD62L APC (Biolegend, clone DREG-56), CD8 PerCP (BD, clone SK1), CD107a FITC (Biolegend, clone H4A3), Ki-67 Pacific Blue (Biolegend clone KI-67), CD69 APC (Biolegend, clone FN50), CD25 APC/Cy7 (Biolegend, clone BC96), CD163 FITC (Biolegend, clone GHI/61), CD54 PE (Biolegend, clone HA58), CD80 PE-Cy7 (Biolegend, clone 2D10) and CD86 APC (Biolegend, clone IT2.2).

Supplementary Figures

Supplemental Figure 1. Transduction levels and killing ability of CD4 and CD8 CAR-T cells

Supplemental Figure 2. Tripartite *in vitro* co-cultures with monocyte-like THP-1 cells

Supplemental Figure 3. Antitumor activity, T-cell expansion and survival rates related to severe CRS in tumor-bearing HuSGM3 mice treated with CD4 and CD8 CAR-T cell products

Supplemental Figure 4. Antitumor activity and survival rates related to severe CRS in tumor-bearing HuSGM3 mice treated with differentially co-stimulated CD4:CD8 1:1 CAR-T cell products

Bove C, *et al. J Immunother Cancer* 2023; 11:e005878. doi: 10.1136/jitc-2022-005878

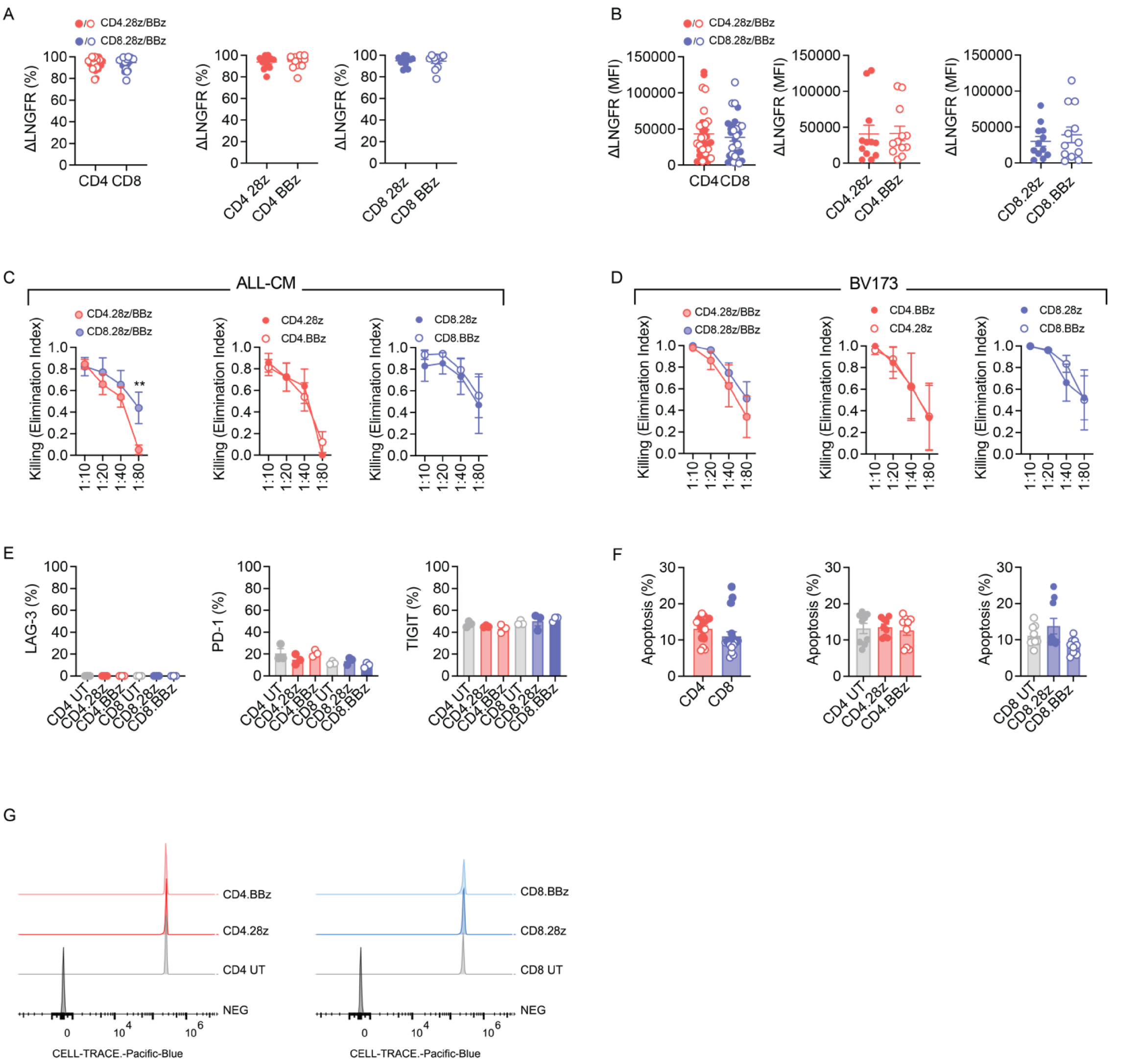

Supplemental Figure 1. Transduction levels, killing ability and tonic signaling of CD4 and CD8 CAR-T cells. ΔLNGFR marker expression reported as (**A**) percentage and (**B**) MFI at the end of manufacturing (n=12). **C)** Killing activity expressed as elimination index (see Methods) and measured by co-culturing CD4.28z (n=3), CD4.BBz (n=3), CD8.28z (n=3) and CD8.BBz (n=3) with CD19+ ALL-CM cells for 4 days at different effector/target (E:T) ratios. **D)** Killing activity measured by co-culturing CD4.28z (n=3), CD4.BBz (n=3), CD8.28z (n=3) and CD8.BBz (n=3) with CD19+ BV173 cells for 4 days at different effector/target (E:T) ratios. **E)** LAG-3, PD-1 and TIGIT exhaustion markers expression at the end of manufacturing (n=3). **F)** Apoptosis analysis at the end of manufacturing (n=3). **G)** Antigen-independent proliferation expressed as Cell-Trace

dilution (n=3). Data are represented as mean ± SEM or mean ± SEM together with overlapping scattered values. By paired t test or 2-way ANOVA.

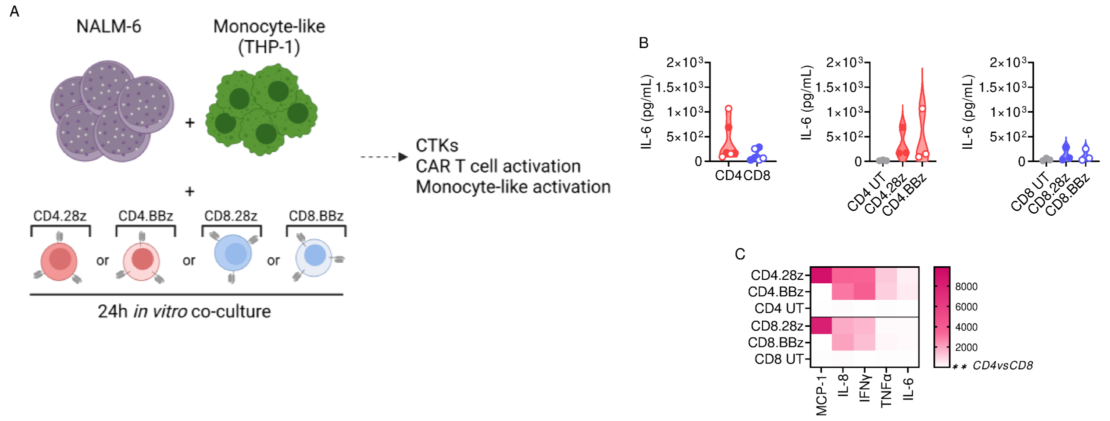

Supplemental Figure 2. Tripartite *in vitro* co-cultures with monocyte-like THP-1 cells. A) Schematic representation of tripartite co-cultures consisting of NALM-6 leukemia cells, CD4 or CD8 CAR-T cells and the monocyte-like THP-1 cell line. **B)** IL-6 production and (**C)** heatmap visualization of cytokine release 24 hours after plating (n=3). Data are represented as mean ± SEM together with overlapping scattered values. **P < 0.01, by paired t test or 2-way ANOVA.

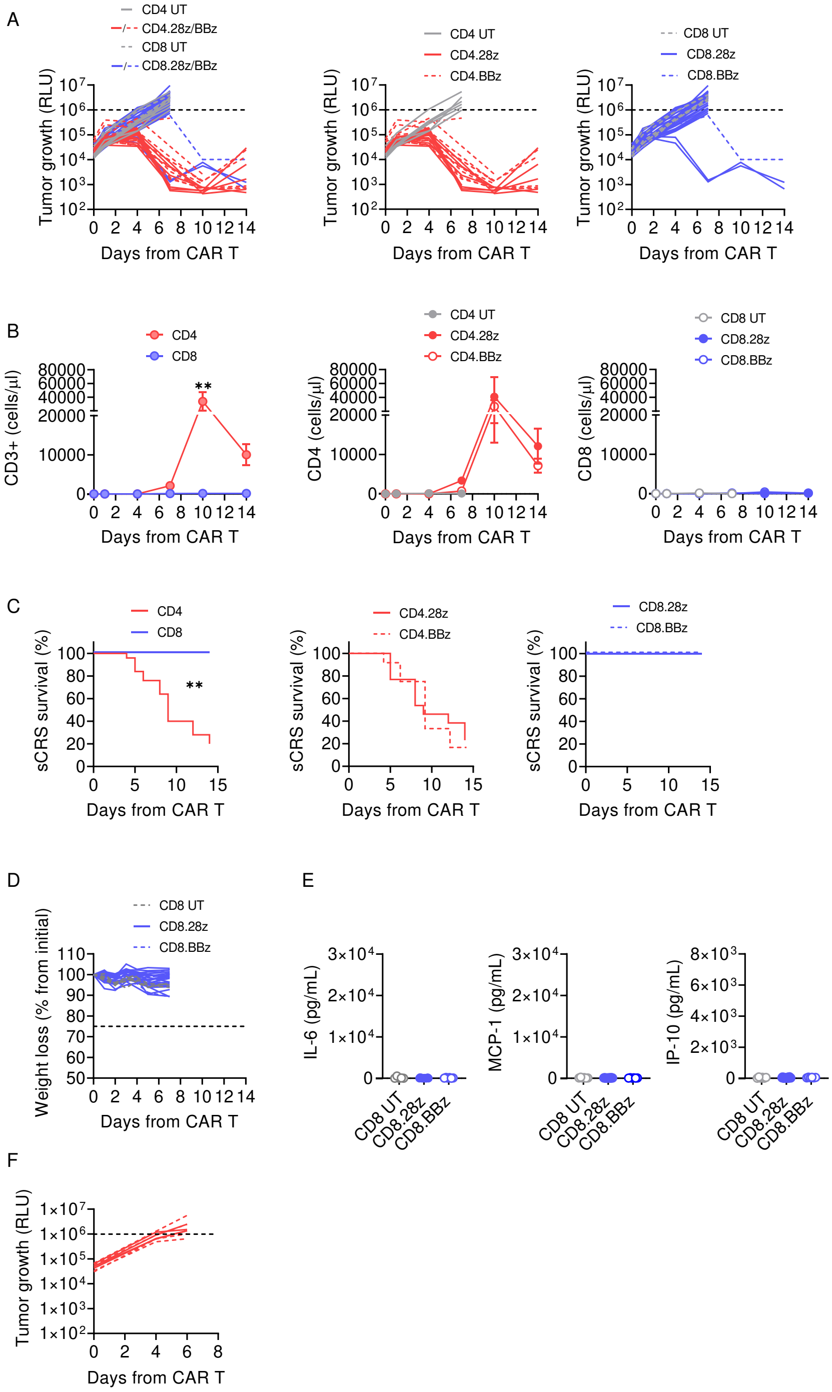

Supplemental Figure 3. Antitumor activity, T-cell expansion and survival rates related to severe CRS in tumor-bearing HuSGM3 mice treated with CD4 and CD8 CAR-T cell products. A-C) Additional data from the experiment reported in Figure 3A-F. **A)**

LUCIA+/NGFR+/NALM-6 bioluminescence signal measured at different time points after treatment with CD4 UT (n=6), CD4.28z (n=13), CD4.BBz (n=12), CD8 UT (n=6), CD8.28z (n=24) and CD8.BBz (n=7) and expressed as relative light units (RLU). **B)** CAR-T cell expansion peak monitored in the peripheral blood of mice receiving CD4 UT (n=6), CD4.28z (n=13), CD4.BBz (n=12), CD8 UT (n=6), CD8.28z (n=2) and CD8.BBz (n=1). **C)** sCRS-related Kaplan-Meier survival analysis of mice treated with CD4.28z (n=13), CD4.BBz (n=12), CD8.28z (n=24) and CD8.BBz (n=7). **D)** Weight loss and **E)** IL-6, MCP-1 and IP-10 serum levels in mice who did not achieve tumor control after treatment with CD8 UT (n=6), CD8.28z (n=22) and CD8.BBz (n=5). **F)** Additional data from the experiment reported in Figure 3G and H. LUCIA+/NGFR+/NALM-6 bioluminescence signal measured at different time points after treatment with CD4.28z (n=4) and CD4.BBz (n=4). Data are represented as mean ± SEM together with individual and overlapping scattered values. **P < 0.01 by 2-way ANOVA, unpaired t test and Mantel-Cox 2-sided log-rank test were performed.

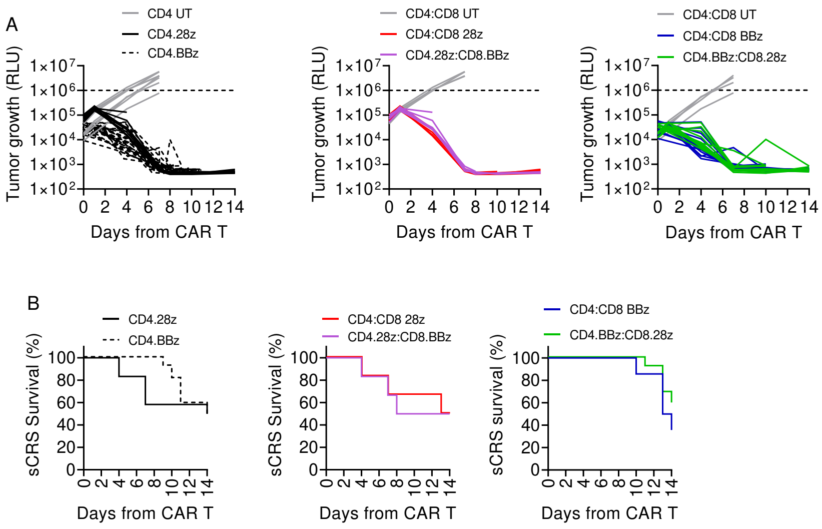

Supplemental Figure 4. Antitumor activity and survival rates related to severe CRS in tumor-bearing HuSGM3 mice treated with differentially co-stimulated CD4:CD8 1:1 CAR-T cell products. Additional data from the experiment reported in Figure 5. **A)** LUCIA+/NGFR+/NALM-6 bioluminescence signal measured at different time points after treatment with CD4:CD8 UT (n=8), CD4:CD8 28z (n= 6), CD4.28z:CD8.BBz (n=6), CD4:CD8 BBz (n= 14) and CD4.BBz:CD8.28z (n=13). **B)** sCRS-related Kaplan-Meier survival analysis of mice. Data are represented as mean ± SEM together with individual scattered values by 2-way ANOVA, unpaired t test, Mantel-Cox 2-sided log-rank test.
